# Whole-Genome Shotgun Sequencing for Nasopharyngeal Microbiome in Pre-school Children With Recurrent Wheezing

**DOI:** 10.3389/fmicb.2021.792556

**Published:** 2022-02-16

**Authors:** Yuping Song, Jinpao Hou, Jamie Sui Lam Kwok, Haoyi Weng, Man Fung Tang, Maggie Haitian Wang, Agnes Sze Yin Leung, Kin Pong Tao, Gary Wing Kin Wong, Renee Wan Yi Chan, Stephen Kwok Wing Tsui, Ting Fan Leung

**Affiliations:** ^1^Department of Pediatrics, Prince of Wales Hospital, The Chinese University of Hong Kong, Sha Tin, Hong Kong SAR, China; ^2^School of Biomedical Sciences, The Chinese University of Hong Kong, Sha Tin, Hong Kong SAR, China; ^3^Jockey Club School of Public Health and Primary Care, Prince of Wales Hospital, The Chinese University of Hong Kong, Sha Tin, Hong Kong SAR, China; ^4^Hong Kong Hub of Paediatric Excellence, The Chinese University of Hong Kong, Sha Tin, Hong Kong SAR, China; ^5^The Chinese University of Hong Kong-University Medical Center Utrecht Joint Research Laboratory of Respiratory Virus and Immunobiology, The Chinese University of Hong Kong, Sha Tin, Hong Kong SAR, China

**Keywords:** dysbiosis, metagenomics, nasopharynx, pre-schooler, wheeze

## Abstract

Microbiome mediates early life immune deviation in asthma development. Recurrent wheeze (RW) in pre-school years is a risk factor for asthma diagnosis in school-age children. Dysbiosis exists in asthmatic airways, while its origin in pre-school years and relationship to RW is not clearly defined. This study investigated metagenomics of nasopharyngeal microbiome in pre-school children with RW. We applied whole-genome shotgun sequencing and human rhinovirus (HRV) detection on nasopharyngeal samples collected from three groups of pre-school children: (i) RW group: 16 children at-risk for asthma who were hospitalized for RW, (ii) inpatient control (IC): 18 subjects admitted for upper respiratory infection, and (iii) community control (CC): 36 children without respiratory syndromes. Sequence reads were analyzed by MetaPhlAn2 and HUMAnN2 algorithm for taxonomic and functional identification. Linear discriminant analysis effect size (LEfSe) analysis was used to identify discriminative features. We identified that *Moraxella catarrhalis* and *Dolosigranulum pigrum* were predominant species in nasopharynx. RW had lower alpha diversity (Shannon diversity index) than CC (0.48 vs. 1.07; *P*_*adj*_ = 0.039), characterized by predominant Proteobacteria. LEfSe analysis revealed *D. pigrum* was the only discriminative species across groups (LDA = 5.57, *P* = 0.002), with its relative abundance in RW, IC, and CC being 9.6, 14.2, and 37.3%, respectively (*P* < 0.05). LEfSe identified five (ribo)nucleotides biosynthesis pathways to be group discriminating. Adjusting for HRV status, pre-school children with RW have lower nasopharyngeal biodiversity, which is associated with Proteobacteria predominance and lower abundance of *D. pigrum*. Along with discriminative pathways found in RW and CC, these microbial biomarkers help to understand RW pathogenesis.

## Introduction

Pre-school age is a critical period for the occurrence of recurrent wheeze (RW) which is a strong risk factor for asthma. Mucosal microbiota serves as a mediator between host immunity and the environment, and microbial dysbiosis was found in many immune-mediated diseases (Kozik and Huang, 2019). Nonetheless, the association between allergies and airway microbiome was underexplored due to limitations with low microbial biomass (Quince et al., 2017). Upper airway samples were widely used in pediatric studies owing to the difficulty in obtaining lower airway samples.

Upper airway dysbiosis is longitudinally related to recurrent wheezing and asthma. Seminal work from Bisgaard et al. (2007) first noticed that asymptomatic hypopharyngeal colonization of pathogenic bacteria including *Streptococcus, Haemophilus*, and *Moraxella* in neonates was related to asthma risk at 5 years old. Similarly, enrichment of *Moraxella*, *Haemophilus*, or *Streptococcus*, in the nasal microbiota from hospitalized bronchiolitis infants was reported being longitudinally related to increased risk for recurrent wheezing (Mansbach et al., 2020; Zhang et al., 2020). Nasal dominance of *Moraxella* and *Staphylococcus* in school-age asthmatic children increased exacerbation risk (McCauley et al., 2019). Nonetheless, there is limited evidence on airway microbiome during acute episodes of RW or asthma exacerbations, particularly in the pre-school age group. Fazlollahi et al. (2018) reported the asthma exacerbation in adult patients is associated with nasal enrichment of *Prevotella*, *Alkanindiges*, and *Gardnerella*. Robinson et al. (2019) conducted pioneering investigation on pre-school children referred for bronchoscope for severe RW, and reported that the dominance of *Moraxella* in bronchoalveolar lavage (BAL) is related to neutrophilic pulmonary inflammation. Nevertheless, BAL microbiome profile was not related to clinical wheezing phenotypes in this study, though a trend of episodic viral wheeze and *Moraxella*-profile was noticed.

Regarding the technological aspect, metagenomics studies are more powerful than 16S rRNA sequencing to delineate microbiome due to their higher resolution and functional profiling capability. A whole genome shotgun (WGS) study reported enriched *Streptococcus pneumoniae* and sphingolipid metabolism pathway in nasopharyngeal aspirate (NPA) to be associated with severe bronchiolitis in infants (Stewart et al., 2017).

We hypothesized that specific markers of nasopharyngeal microbiome (NPM) from pre-school wheezers at risk of developing asthma can discriminate them from non-wheeze inpatient and community controls (CCs). Besides, we hypothesized that human rhinovirus (HRV) infection might influence the association between NPM and pre-school wheezing. This study applied WGS to characterize the NPM in pre-school children hospitalized for RW who were positive for asthma predictive index (API), and explore the differences in NPM compositions and functional capabilities between RW cases and control groups and the possible effect of HRV infection on such associations between NPM and pre-school wheezing.

## Materials and Methods

### Subjects

Three groups of Chinese pre-school children aged 2–5 years old were recruited: (a) children hospitalized for RW who were positive for stringent-asthma predictive index (S-API) (Castro-Rodríguez et al., 2000); (b) inpatient control (IC) children hospitalized for upper respiratory tract infection (URTI) with no respiratory distress, normal auscultation and chest radiograph, and no history of wheeze and asthma. These IC subjects were matched for gender and admission time (hospitalized within 1 week from the RW case recruitment) with RW patients; (c) CC children from nurseries and kindergartens who participated in our influenza surveillance study (Leung et al., 2017). These subjects were free from URTI for ≥ 4 weeks and had no history of asthma, allergic rhinitis, eczema, and food allergy. All subjects must not receive antibiotics within 4 weeks before study. Chinese University of Hong Kong – New Territories East Cluster Clinical Research Ethics Committee approved this study.

Following informed consent, NPA was collected from hospitalized subjects (RW and IC) upon admission; flocked nasopharyngeal swab (FNPS; Copan Diagnostics, Corona, CA, United States) was obtained from CC subjects at the kindergartens. All samples were placed immediately at 4^°^C in 2 ml viral transport medium containing 0.1 mg/ml gentamicin, 500 IU/ml penicillin, 500 IU/ml streptomycin, and 2.5 μg/ml fungizon. As HRV was the most important trigger for RW (Leung et al., 2010), 50 μl of each sample was aliquoted for HRV detection by molecular assays according to our published method (Mak et al., 2011). The remaining portion was stored at -80^°^C until microbiome analysis in one batch.

### DNA Extraction and Library Preparation

Total genomic DNA was extracted from 300 μl of respiratory secretion using MO BIO PowerSoil DNA Isolation Kit (Mo Bio Laboratories; Carlsbad, CA, United States). The concentration and purity of extracted DNA was measured by NanoDrop^®^ ND-2000 (Thermo Fisher Scientific, Waltham, MA, United States), with all samples having ≥ 20 ng DNA and acceptable OD260/280 ratio. DNA integrity was confirmed by 1% agarose gel electrophoresis (120 V, 45 min).

### High-Throughput Sequencing and Sequence Data Pre-processing

Whole genome shotgun sequencing was performed by Groken Bioscience (Hong Kong) on HiSeq X Ten platform (Illumina, San Diego, CA, United States) to generate paired-end outputs (2 × 150 bp). The raw reads were pre-processed using KneadData version 0.7.2 for quality control and host sequence decontamination (Bowtie2 version 2.3.4.3 with human reference genome GRCh37/hg19). Please refer to online supplement for the details.

### Taxonomic and Functional Analysis

Taxonomy assignment and functional prediction were performed using MetaPhlAn2 version 2.7.7 and HUMAnN2 version 0.11.2 with default settings. Based on the reference of clade-specific marker genes embracing viruses, archaea, bacteria, and eukaryotes, MetaPhlAn2 classifies reads belonging to these kingdoms down to species level (Truong et al., 2015). HUMAnN2 is a three-tire pipeline designed for annotating the functional capabilities of microbes from metagenomic datasets based on MetaCyc database (Franzosa et al., 2018). Results were visualized by R version 3.5.0 (see online supplement).

### Statistical Analysis

To determine the differences in clinical variables across groups, Wilcoxon rank-sum test were used for comparing categorical variables between two groups; Kruskal–Wallis test and Chi-square test were performed for comparison of continuous and categorical variables across more than two groups, respectively. Alpha diversity represented by Shannon diversity index (SDI) was compared across multiple groups using Kruskal–Wallis test. NPM composition (beta diversity) were compared using PERMANOVA adonis test in vegan R package with 999 permutations. Regarding taxonomic and functional profiles, Statistical Analysis Metagenomic Profiles (STAMP) version 2.0 (Parks et al., 2014) was used to compare between-group differences. Unless stated otherwise, pairwise comparison between groups were performed using Dunn’s *post hoc* test coupled with Benjamini–Hochberg false discovery rate (FDR) procedure for multiple testing correction. Linear discriminant analysis effect size (LEfSe) algorithm was used to identify discriminating features that were significant both statistically and biologically (biomarker discovery). Logarithmic LDA score > 2 was set as the threshold to estimate effect size. Considering the complexity of metagenomic profiles, we replicated findings from LEfSe by analysis of composition of microbiomes (ANCOM) (Mandal et al., 2015) which has sensitivity on complexed microbiome profile and advantage of low FDR (see online supplement). Considering possible impact of HRV infection and on microbiota (Molyneaux et al., 2013; Korten et al., 2016), HRV status was included as confounders in our data analysis. This study considered potential microbial biomarkers microbes or their metabolic pathways that displayed consistent statistical significance from all three analyses above and remained group-discriminating after HRV adjustment. *P* < 0.05 was set as the significance threshold.

## Results

### Subjects

Nasopharyngeal microbiome profiles were successfully recovered from a total of 70 samples, including 16 RW, 18 IC, and 36 CC. Eleven subjects (8 for RW and 3 IC) were excluded as non-human metagenomic reads and their samples were deemed “100% unclassified” after WGS analysis. Supplementary Table 1 summarizes clinical characteristics of analyzable study participants. We found that the age of CC subjects (median age: 5.0 years old) is higher than IC (median 3.3 years, *P_*adj*_* < 0.01) and RW (median 3.5 years, *P_*adj*_* < 0.01) groups. All RW samples (RWV) were positive for HRV (HRV+), whereas HRV was detected in 33.3% (6/18) of IC and 22.2% (8/36) of CC. These two control groups were further divided into ICV (positive for HRV; *n* = 6), ICC (negative for HRV; *n* = 12), CCV (positive for HRV; *n* = 8), and CCC (negative for HRV; *n* = 28) subgroups.

### The Community Controls Had Higher Nasopharyngeal Microbiome Biodiversity Than Patients Hospitalized for Recurrent Wheezing

**FIGURE 1:**
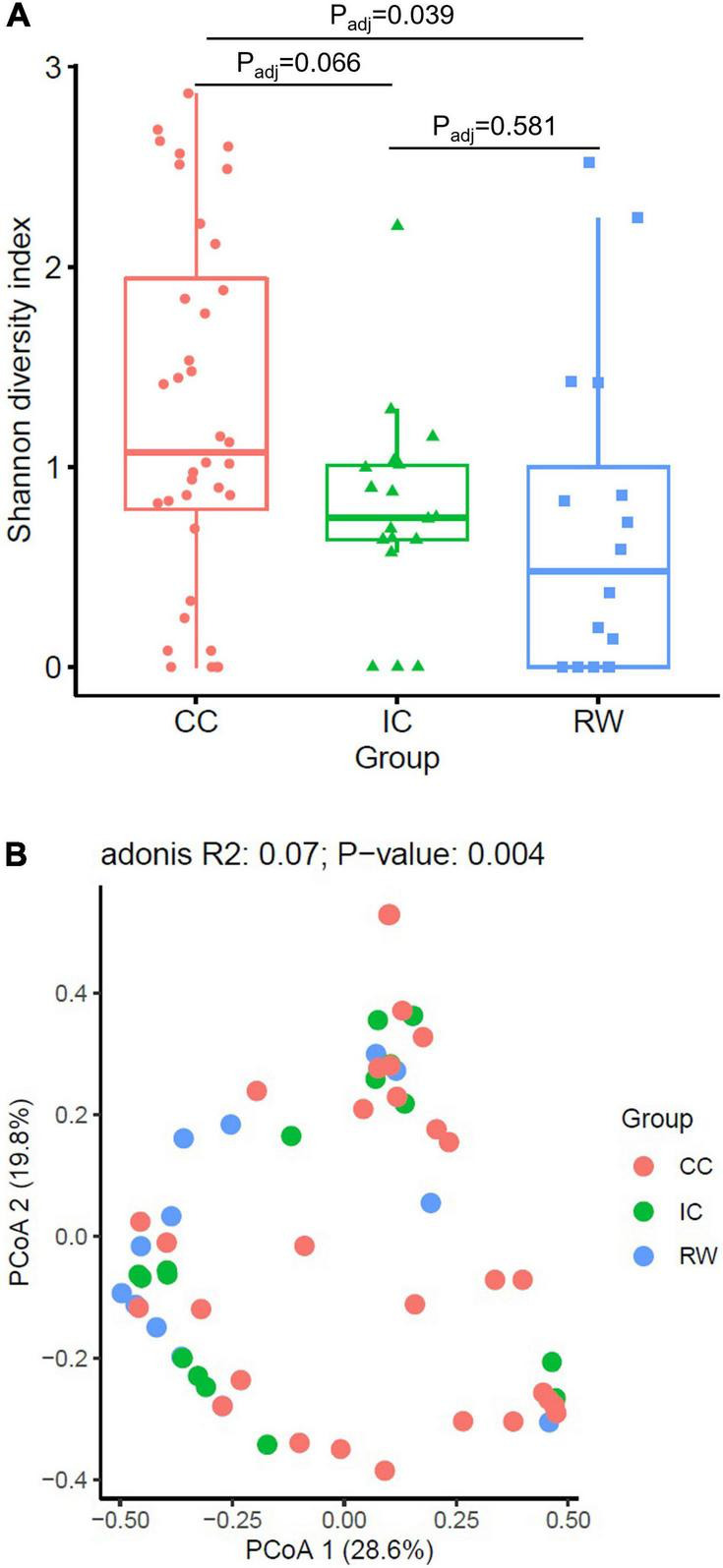
Alpha and beta diversity of NPM in subjects from three clinical groups. **(A)** Boxplots of Shannon diversity index among RW (*n* = 16), IC (*n* = 18) and CC (*n* = 36) groups. **(B)** Principal coordinate analysis (PCoA) plot based on Bray–Curtis dissimilarity. PERMANOVA test were performed by adjusting for age and HRV status. Points refer to samples that were color coded by each group. CC, community control; IC, inpatient control; RW, recurrent wheeze.

Whole genome shotgun yielded an average throughput of 14 million 150-bp raw reads per sample, among which 98% passed quality filtering using standard parameters and > 90% of read bases met the Q30 criteria. Approximately 0.1–0.3 million pairs of reads (1.4–4%) considered non-human were subjected to downstream analysis. Since SDI was positively correlated with age (Spearman *R* = 0.29, *P* = 0.016), age was a confounder of the relationship between RW and SDI. The median SDI for RW, IC, and CC groups were 0.48, 0.75, and 1.07 respectively (Figure 1A), with SDI being higher in CC than RW (Benjamini–Hochberg FDR, *P_*adj*_* = 0.039) after adjustment for age. This difference between subgroups remained significant after adjustment for HRV status (Kruskal–Wallis test, *P* = 0.048, Supplementary Figure 1A). However, no significant difference in SDI was detected by pairwise comparisons between subgroups (*P* > 0.05 for all). This was consistent with our finding that SDI was not associated with HRV status (*P* = 0.191).

### Discriminative Taxonomic Profiles in Clinical Groups

Based on Bray–Curtis dissimilarity, we observed that NPM composition was associated with HRV status (PERMANOVA, *R*^2^ = 0.052, *P* = 0.003) but not with age (*R*^2^ = 0.015, *P* = 0.446). PERMANOVA analysis indicated that NPM composition in CC group is different from that of IC group (*R*^2^ = 0.06, *P_*adj*_* = 0.006) and RW group (*R*^2^ = 0.067, *P_*adj*_* = 0.006) after controlling for HRV status (Figure 1B). Furthermore, subjects in CC who were negative for HRV infection (CCC) had distinct NPM structure compared to those in IC with HRV infection (*R*^2^ = 0.096, *P_*adj*_* = 0.02 for RWV; *R*^2^ = 0.095, *P_*adj*_* = 0.035 for ICV; Supplementary Figure 1B).

**FIGURE 2:**
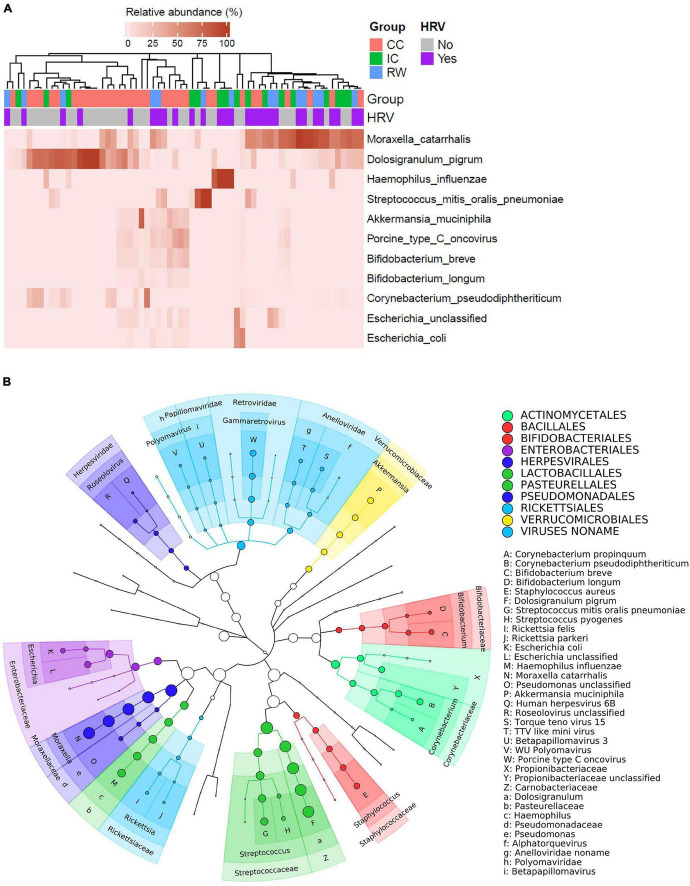
Taxonomic profiles of NPM in all 70 subjects. **(A)** Heatmap showing relative abundances of the top 11 most abundant species in individual samples. Samples were hierarchically clustered based on Bray–Curtis dissimilarity of species abundances, and color coded according to group or HRV status as indicated. **(B)** Taxonomic cladogram illustrating the phylogenetic diversity of microbial species (bacteria and viruses) found in all samples. Size of the node was proportional to the relative abundance for the corresponding taxon. Family and genus-level annotations were labeled on the tree, with some of which annotated using X–Z and a–i keys to avoid image overlapping. Species with minimal relative abundance threshold of 1% were annotated using A–W as keys. Order-level microbes are indicated by different colors shown in the legend.

At the species level, WGS identified 28 bacteria, 12 viruses, and one fungus (*Aspergillus fumigatus*). Supplementary Figure 2 shows relative abundance (RA) of all identified microbes in all 70 samples in each kingdom. The 90th percentile RA across all samples for each species was calculated and ranked to identify the topmost abundant species, which is the default parameter implemented in MetaPhlAn2. Supplementary Table 2 summarizes the top 11 most abundant species, and Figure 2A illustrates heatmap of their RA, in which samples were hierarchical clustered based on Bray–Curtis dissimilarity. *Moraxella catarrhalis* and *Dolosigranulum pigrum* were the predominant species in our nasopharyngeal samples, with RA > 70% being found in ∼10% of our samples. Figure 2B shows the cladogram on phylogenetic relationship among the presenting species. Of note, among 12 viruses detected by WGS, *porcine type C oncovirus* ranked the third based on 90th percentile of RA and was predominantly detected in CC group (Supplementary Figure 3; *P* = 0.044).

Thirty-three species [21 bacteria, 11 viruses, and one eukaryote (*Aspergillus fumigatus*)] had different RA among three groups (Supplementary Table 3). Among these species, five bacteria and one virus had an eta squared effect size (η^2^) > 0.06, suggesting medium to high biological relevance to the clinical diagnosis (Table 1; Watson, 2018). After adjusting for HRV status, *D. pigrum* was the only species that remained group-discriminating in LEfSe analysis (Supplementary Figure 4). Because bacteria accounted for nearly 90% of non-human reads, subsequent taxonomical and functional analyses focused on bacterial sequences. LEfSe revealed seven microbial taxa as significant in discriminating between groups (LDA score > 2, *P* < 0.05; Supplementary Figure 5A). Supplementary Figure 5B depicts their phylogenetic relationship. Proteobacteria was the only phylum representative for RW (LDA score 5.13, *P* = 0.026), although we did not find any discriminative genus or species within this phylum. In contrast, *D. pigrum*, *GCF_000245815* (*the strain ATCC 51524 belonging to D. pigrum*), the family Carnobacteriaceae (*Dolosigranulum*), and the phylum Actinobacteria, were identified as the discriminative taxa in CC. Adjusted for HRV status, *D. pigrum* was the only discriminative species across three groups with lower abundance in RW and IC than in CC (Supplementary Figure 5C).

**TABLE 1:** Microbial species with medium to high effect size across different subject groups identified by STAMP.

Species	Relative abundance			*q*-value	η^2^ effect size
	*RW*	*CC*	*IC*		
*Dolosigranulum pigrum*	6.35 ± 16.80	34.66 ± 34.49	12.24 ± 22.21	0.0008	0.18
*Bifidobacterium longum*	0.25 ± 0.88	1.47 ± 2.74	0.02 ± 0.11	0.013	0.11
*Bifidobacterium breve*	0.78 ± 2.58	3.89 ± 7.49	0.11 ± 0.49	0.014	0.10
*Akkermansia muciniphila*	0.89 ± 2.98	5.86 ± 14.34	0.11 ± 0.50	0.029	0.07
*Corynebacterium pseudodiphtheriticum*	0.80 ± 3.46	5.64 ± 13.14	0.87 ± 2.28	0.029	0.06
*Porcine type C oncovirus*	1.23 ± 4.11	5.87 ± 11.43	0.36 ± 1.62	0.015	0.09

**FIGURE 3:**
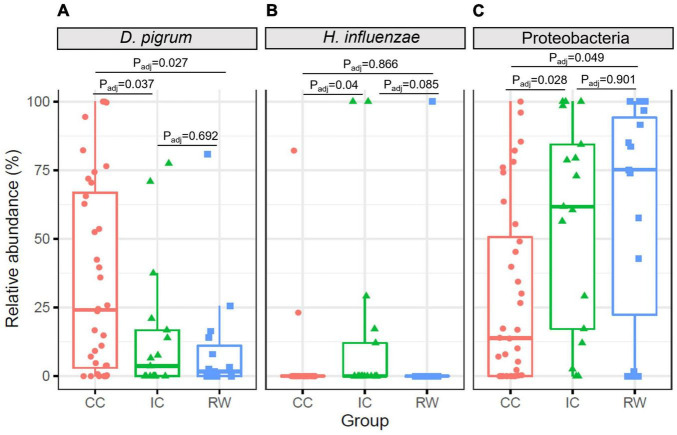
Boxplots showing relative abundances of differentially abundant taxa at different taxonomic levels identified by ANCOM. **(A)** Left panel, *D. pigrum* species had higher abundance in CC group but lower abundance in IC and RW groups. **(B)** Middle panel, *H. influenzae* was more abundant in IC than CC group. **(C)** Right panel, *Proteobacteria* was more abundant in RW and IC than CC. CC, community control; IC, inpatient control; RW, recurrent wheeze.

To confirm the above findings, ANCOM was performed on bacterial taxa that were present in ≥ 10% of all samples. *D. pigrum* and *Haemophilus influenzae* as well as Proteobacteria phylum were differentially abundant between cases and controls (*P* < 0.05; Figures 3A–C). Specifically, *D. pigrum* had a higher RA in CC (37.3 ± 35.3%) compared to IC (14.2 ± 24.5%, *P_*adj*_* = 0.037) and RW (9.6 ± 21%, *P_*adj*_* = 0.027, Benjamini–Hochberg FDR). *H. influenzae* was more abundant in IC (14.6 ± 32.9%) than the other two groups (6.3 ± 25.8% in RW and 3.0 ± 14.1% in CC). Consistent with LEfSe analysis, Proteobacteria was more abundant in RW than CC (*P* = 0.049). After adjusting for HRV status and age, *D. pigrum* (IC vs. CC, *P_*adj*_* = 0.042) and Proteobacteria (IC vs. CC, *P_*adj*_* = 0.022) remained differentially abundant across samples (Supplementary Figures 6A,C), while that of *H. influenza* became insignificant. Interestingly, *H. influenza* was primarily detected in HRV-positive IC samples (Supplementary Figure 6B), suggesting this species may contribute to URTI-related hospitalization.

### Functional Potential of Nasopharyngeal Microbiome

**FIGURE 4:**
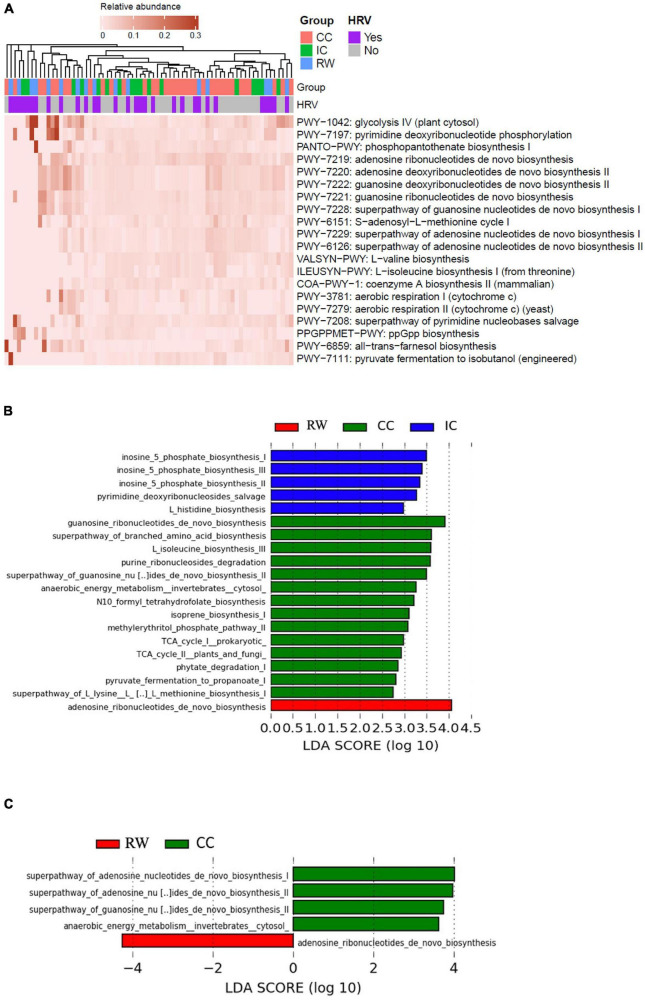
Functional characterization of NP microbiome in control and RW subjects. **(A)** Heatmap of the top 20 most abundant MetaCyc pathways in individual samples as characterized by HUMAnN2. Samples were hierarchically clustered based on Bray–Curtis dissimilarity of pathway abundances, and color coded according to group or HRV status as indicated. **(B)** Histogram of discriminative pathways across all samples as identified by LEfSe. Kruskal–Wallis test was performed with clinical features as the only grouping factor at the thresholds of LDA score > 2 and *P* < 0.05. **(C)** Histogram of discriminative pathways across all samples as identified by LEfSe in which clinical features and HRV status were analyzed as “class” and “subclass” factors respectively. RW, recurrent wheeze (red); IC, inpatient controls (blue); and CC, community controls (green).

HUMAnN2 recognized a total of 301 pathways. Supplementary Table 4 summarizes the top 20 most abundant pathways (ranked by 90th percentile of RA across all samples), whereas Figure 4A illustrates the heatmap of their corresponding RA in individual samples. Purine deoxyribonucleotide *de novo* biosynthesis (3.07 ± 3.05% for both adenosine and guanosine, respectively) and glycolysis IV (5.3 ± 1%) were the topmost abundant pathways having the highest 90th percentile of RA. STAMP analyses identified 32 pathways to be significantly different (corrected *P* < 0.05) with a moderate to high eta-squared effect size (η^2^ > 0.06) across groups (Supplementary Table 5). Seven of these 32 significant pathways were also among the top 20 most abundant: glycolysis IV (plant cytosol), superpathway of adenosine nucleotides *de novo* biosynthesis II, S-adenosyl-L-methionine cycle I, guanosine ribonucleotides *de novo* biosynthesis, superpathway of guanosine nucleotides *de novo* biosynthesis I, superpathway of adenosine nucleotides *de novo* biosynthesis I and L-valine biosynthesis. Hierarchical clustering of samples according to pathway abundances showed a clear pattern that PWY-1042: glycolysis IV (plant cytosol) (*q*-value = 0.048, η^2^ = 0.11) and GLYCOGENSYNTH-PWY: glycogen biosynthesis I (from ADP-D-glucose) (*q*-value = 0.044, η^2^ = 0.06) were the only two pathways being enriched in RW. By contrast, purine metabolism such as guanosine ribonucleotides *de novo* biosynthesis (PWY-7221, *P_*adj*_* = 0.02, η^2^ = 0.12), and superpathway of guanosine nucleotides *de novo* biosynthesis I (PWY-7228, *P_*adj*_* = 0.025, η^2^ = 0.09) pathways were significantly more abundant in CC (PWY-7221, 3.26 ± 3.3%; PWY-7228, 2.88 ± 2.65%) as compared to IC (PWY-7221, 1.51 ± 0.95%; PWY-7228, 1.66 ± 1.08%) and RW (PWY-7221, 1.42 ± 1.7%; PWY-7228, 1.48 ± 1.87%) groups.

When analyzed by LEfSe (Figure 4B), RW was enriched in adenosine ribonucleotides *de novo* biosynthesis (LDA = 4.06, *P* = 0.006). Bacterial enzymes involved in inosine 5 phosphate biosynthesis were more abundant in IC than the other groups (LDA = 3.49, *P* < 0.001) whereas guanosine ribonucleotides *de novo* biosynthesis was enriched in CC (LDA = 3.91, *P* = 0.004). Fifteen of these 20 pathways overlapped with those identified using STAMP, and the remaining five pathways also belonged to three categories mentioned above (i.e., Biosynthesis, Degradation/utilization/assimilation, and Generation of Precursor Metabolite and Energy). Adjusting for HRV status, five discriminative MetaCyc pathways remained significant even by using the more stringent “all-against-all” strategy in multi-class comparison implemented in LEfSe (Figure 4C). Adenosine ribonucleotides *de novo* biosynthesis (MetaCyc pathway PWY-7219) was the only representative pathway in RW (LDA = 4.25, *P* = 0.006), while four discriminative components were found in CC (Table 2).

**TABLE 2:** Discriminative pathways for RW and CC groups as identified by LEfSe analysis.

Pathway	Group discriminated	*q*-value	LDA score
PWY-7219: adenosine ribonucleotides *de novo* biosynthesis	RW	0.006	4.25
PWY-7229: superpathway of adenosine nucleotides *de novo* biosynthesis I	CC	0.012	4.03
PWY-6126: superpathway of adenosine nucleotides *de novo* biosynthesis II	CC	0.020	3.98
PWY-6125: superpathway of guanosine nucleotides *de novo* biosynthesis II	CC	0.014	3.75
PWY-7383: anaerobic energy metabolism (invertebrates, cytosol)	CC	0.001	3.63

*LEfSe analysis was performed by Kruskal–Wallis test and pairwise Wilcoxon test.*

### Linking Metabolic Pathways to Microbes

To understand the species contributing to a particular functional pathway, contributional diversity bar plots illustrating the linkage between metabolic pathway and attributable species were generated for the five discriminative pathways (Supplementary Figure 7). The discriminative pathways representing CC group (i.e., PWY-7229, PWY-6126, and PWY-6125), being present only in a small number of samples, were contributed mainly by *Streptococcus spp.* and *H. influenzae* (Supplementary Figures 7A–C). In contrast, PWY-7219 being enriched in RW was present in 82.9% of all samples. This pathway was contributed mainly by *M. catarrhalis* in RW and IC groups and by *D. pigrum* and *Akkermansia muciniphila* in the CC group (Supplementary Figure 7D). This result was consistent with our previous finding of over-representation of *M. catarrhalis* in RW and *D. pigrum* in CC. To further dissect the functional potential of *D. pigrum*, we identified nine MetaCyc pathways in which *D. pigrum* was involved (Supplementary Table 6). The contributional bar plots revealed that, compared to RW and IC groups, S-adenosyl-L-methionine cycle I (PWY-6151) and purine ribonucleosides degradation (PWY0-1296) were more abundant in CC and mainly contributed by *D. pigrum* (Supplementary Figure 8).

Of interest, several pathways identified from our dataset were related to genes from other kingdoms. Specifically, we found pathways specific to *protozoa* and *fungi* among the control subjects. These pathways included PWY-7383: anaerobic energy metabolism (invertebrates, cytosol) (*q* = 0.013, η^2^ = 0.09) and PWY-5690: TCA cycle II (plants and fungi) (*q* = 0.025, η^2^ = 0.07) in CC group, and PWY-7357: thiamin formation from pyrithiamine and oxythiamine (yeast) (*q* = 0.038, η^2^ = 0.07) in IC group (Supplementary Table 5). Besides, the previously mentioned pathway PWY-1042 enriched in RW is a glycolysis pathway contributed by *Viridiplantae* kingdom.

## Discussion

This WGS-based study revealed that *M. catarrhalis* (Proteobacteria phylum) and *D. pigrum* (Firmicutes phylum) were predominant species in nasopharyngeal samples of pre-school children. This was concordant with other studies on NPM among infants and children (Bogaert et al., 2011; Teo et al., 2015). We discovered that the NPM of RW with asthma predisposition was characterized by low biodiversity characterizing with enriched phylum Proteobacteria being the only representative taxa. Conversely, *D. pigrum* was overrepresented in CC. Adjusted for HRV status, *D. pigrum* was the only discriminative species across RW, IC, and CC groups. LEfSe analyses identified five discriminative MetaCyc pathways, including adenosine ribonucleotides *de novo* biosynthesis in RW and four bacterial pathways involved in purine and adenosine biosynthesis, and energy metabolism for CC group.

In the present study, two approaches were applied for collecting nasopharyngeal samples from hospitalized and community subjects. For hospitalized patients, NPAs were obtained as standard practice for pathogen detection upon admission (Leung et al., 2010). On the other hand, FNPSs were collected from CC subjects as such approach (versus NPA) obviates the need for negative-pressure facilities during the procedure (Chan et al., 2008). In practice, Tunsjø et al. (2015) reported that these two sample types did not differ in virus detection among children. Previous studies have also indicated that FNPS samples represented a less invasive and more reliable option than NPA in terms of the sensitivity and specificity for respiratory virus detection especially for children below 5 years old (Abu-Diab et al., 2008; Lambert et al., 2008). These findings supported our use of FNPS in CC subjects and NPA for inpatients and would not introduce any substantial difference in nasopharyngeal microbial communities from the methodological point of view.

Our study reported that hospitalized children with RW (and positive S-API) or URTI had lower microbial biodiversity in upper airway than CC. Specifically, pre-school children with RW had higher abundance of Proteobacteria while the latter group possessed higher abundance of phylum Actinobacteria and genus *Dolosigranulum* in their nasopharynx. These observations were consistent with previous studies for wheeze and asthma that the asthmatic upper airway had lower biodiversity but more abundant Proteobacteria (Depner et al., 2017; Fazlollahi et al., 2018). Particularly, *Moraxella* is a genus under Proteobacteria that was repeatedly reported to be related to asthma and wheeze. Children hospitalized for severe bronchiolitis who carried more *Moraxella* in upper airway had increased risk for RW at later childhood (Mansbach et al., 2020; Zhang et al., 2020). Similar observations between nasal *Moraxella* and asthma exacerbation were found in schoolchildren (McCauley et al., 2019; Zhou et al., 2019). McCauley et al. (2019) reported nasal domination of *Moraxella spp.* was related with type-2 inflammation in nasal epithelium. All our RW cases were positive for S-API which reflected familial and personal history of allergic diseases. Further investigation is warranted to link the host inflammatory profile to NPM dysbiosis during acute wheezing episodes.

Moreover, Robinson et al. reported that pre-school episodic viral wheezers had *Moraxella-*dominating lung microbiome during the stable phase. Their results also suggested that *Moraxella*-enriched microbial profile was related to local but not systemic neutrophilic inflammation (Robinson et al., 2019). Our study extended the relationship between Proteobacteria and wheezing illnesses into pre-school age group. Additionally, our finding supported that NPM dysbiosis shared similar patterns with that found in BAL from severe pre-school wheezers. This observation was consistent with a previous study that upper airway microbiota in young children was generally representative for lung disease-related microbial patterns in the lower airway (Marsh et al., 2016).

On the contrary, Actinobacteria and *Dolosigranulum* might protect against asthma (Biesbroek et al., 2014; Ta et al., 2018). Both taxa were normal upper airway commensals that colonized infants’ nasopharyngeal niche within the first month of life (Bosch et al., 2017; Man et al., 2017). The predominance of *Dolosigranulum* in the airway was associated with lower risk for asthma and viral respiratory diseases (Bosch et al., 2017; Teo et al., 2018). *Dolosigranulum* and *Corynebacterium* (Actinobacteria) also played an important role in respiratory health (Laufer et al., 2011; Kozik and Huang, 2019). Our study extended such relationship into the pre-school age group. Our results suggested that RW-related dysbiosis in the upper airway was present prior to the onset of asthma among pre-school children. This finding may provide an investigative approach for asthma prediction in young children.

The ability of WGS sequencing to taxonomically identify microbes down to the species level (e.g., *M. catarrhalis* in RW and *D. pigrum* in CC) is one of its strengths over 16S rRNA sequencing. Our metagenomic data identified that *D. pigrum* was the only species that remained group-discriminating after adjustment for HRV status. This bacterium was first described as a new lineage of lactic acid bacterium (Aguirre et al., 1993), and its whole-genome sequence was announced in 2017 (Mukhopadhyay et al., 2017). Recent studies revealed an inverse correlation in RAs between *D. pigrum* and *Staphylococcus aureus*, a pathobiont in the respiratory tract (Liu et al., 2015; Escapa et al., 2018). Furthermore, *D. pigrum* was reported to inhibit the *in vitro* growth of *S. aureus* (Brugger et al., 2020) and decrease the production of epithelial pro-inflammatory cytokines induced by *S. aureus* which maintain epithelial barrier integrity against disruptive interleukin-4 (De Boeck et al., 2017).

In addition, another strength of WGS is its capability to identify all kingdoms of microbes that use DNA as their genetic materials while 16S rRNA profiling could only reconstruct phylogeny of bacteria and archaea. This WGS study uncovered six viral families including *Siphoviridae*, *Herpesviridae, Anelloviridae, Papillomaviridae, Polyomaviridae*, and *Retroviridae* from nasopharyngeal samples that accounted for nearly 10% of non-human reads. Among them, *Herpesviridae*, *Polyomaviridae*, and *Anelloviridae* were reported in children with community-acquired pneumonia (Li Y. et al., 2019). We found *porcine type C oncovirus* of the Retroviridae family to be more abundant in CC (Supplementary Figure 3), and existing data suggested low risk of *in vivo* infection of this virus in human (Łopata et al., 2018).

Another remarkable advantage of WGS related to its ability to characterize the functional potential of a metagenome. Accumulating evidence indicated that mucosal microbiome impacted allergy and asthma development through perturbation of the host immunity (Kemter and Nagler, 2019). We found a higher functional capacity for purine nucleotide *de novo* biosynthesis (PWY-7229, PWY-6126, and PWY-6125) in CC subjects (Table 2), suggesting nasopharyngeal microbes with greater proliferation potential could contribute to immune homeostasis in healthy individuals. Nevertheless, these discriminative metabolic pathways were present in only less than one quarter of all CC samples (Supplementary Figures 7A–C). Hence, the immunological implications of overrepresentation of these pathways required further investigation integrating metatranscriptomics and metabolomics. In this regard, Stewart et al. (2017) interrogated the microbiome and metabolome in NPA and explored their relationship to bronchiolitis in infants. In school-age children with asthma, serum metabolomic biomarkers were linked to oropharyngeal microbiome to illustrate the interaction between microbes and host immunity (Chiu et al., 2020). Similar exploration leveraging multi-omics approaches are warranted to implement in pre-school children with recurrent wheezing, which would further our understanding on how functionally aberrant airway microbiome modulates host immune response during this critical phase and leads to later asthma.

As discussed above, *D. pigrum* has been shown to inhibit *in vitro* growth of *S. aureus* (Brugger et al., 2020). Among the nine *D. pigrum-*involved pathways, PWY-7219 represented the only discriminative function enriched in RW by LEfSe analysis (and HRV status as a subclass grouping factor). Interestingly, this pathway was mainly contributed by *M. catarrhalis* in RW but primarily by *D. pigrum* in CC (Supplementary Figure 7D). S-adenosyl-L-methionine cycle I (PWY-6151) responsible for amino acid metabolism was another *D. pigrum*-involved pathway. By providing methyl in living cells (Caspi et al., 2017), this pathway was important in the biosynthesis of multiple antibiotics including neomycin B (Kudo et al., 2014) and anthracycline (Grocholski et al., 2015), which might partially explain inhibition of *S. aureus* growth by *D. pigrum* in co-culture. Moreover, some asthmatic children present with chronic cough (Morice et al., 2014). Of interest, lung-derived ATP interacted with P2X receptor (evolutionarily conserved ATP-gated ion channels) to stimulate hypersensitive cough through peripheral nerves (Turner and Birring, 2019). Endogenous ATP could be produced locally in response to tissue stresses such as inflammation or epithelial damage (Ford and Undem, 2013). Our metagenomic data revealed that the NPM from CC group was enriched for purine and adenosine biosynthesis. Whether this finding may be related to ATP-P2X pathway require further investigation.

Persistent bacterial bronchitis (PBB) is another leading cause of chronic cough in young children. Differentiating these two conditions is important as the treatment plan and prognosis are distinct. Kazachkov et al. (2018) investigated microbiome of BAL samples from pre-school children with chronic cough. They found that asthmatic BAL was enriched for *Rothia, Gemellaceae*, and *Granulicatella*, while that of PBB was characterized by increased abundance for *Prevotella*. Another study reported bronchial dysbiosis in pre-school PBB to be associated with enrichment of *Haemophilus* and *Neisseria* when compared to healthy controls (Cuthbertson et al., 2017). Similarly, Bao et al. (2018) extended this finding to infants by showing that those with PBB had enriched *Haemophilus* and *Bacteroides* but decreased *Lactococcus* and *Lactobacillus*.

Some studies suggested that upper airway samples, such as throat swabs, demonstrated representative microbial communities for lower airways as assessed by BAL or bronchial brushes (Ahmed et al., 2018). This finding supported the use of upper airway samples for studying microbiome in different respiratory diseases in children as it would be too invasive to obtain BAL samples by bronchoscopy in most cases. Studies using upper airway surrogates are warranted to unveil potential microbial signals for early recognition of asthma-predisposed signals among children with chronic cough.

Of interest, this study identified pathways (PWY-7383, PWY-5690, and PWY-7357) specific to *fungi* among the control subjects. This supported the hypothesis that fungi in NPM may play a role in the pathogenesis of recurrent wheezing (Li E. et al., 2019). Fujimura et al. (2016) reported higher RAs of specific fungi (*Candida* and *Rhodotorula*) in the gut pose a high risk of childhood asthma. Fungal dysbiosis characteristic of *Pneumocystis* overrepresentation in the lung was associated with severe asthma in children (Barcik et al., 2020). More studies focusing on airway fungiome are needed to deepen our understanding of the mechanisms through which fungi influence respiratory health.

There are several limitations in this study. Firstly, we could not analyze the effect of HRV on RW as all our RW cases were HRV positive. Our local findings found that the large majority of hospitalized children with RW and asthma exacerbation had HRV infection (Leung et al., 2010; Mak et al., 2011). However, we were still able to adjust for HRV by having HRV-negative subjects in the IC and CC groups. Secondly, this was a cross-sectional study on pre-school recurrent wheezers which attempted to apply NPM to predict later development of asthma. The results will need to be validated in prospective cohorts that followed RW group into later childhood. In addition, WGS is able to reveal the metabolic potentials embedded in the microbial genes. The integration of multi-omics information allows us to explore the mechanisms through which microbial dysbiosis affects respiratory health, but *in vitro* and animal experiments are required to confirm the functionality of these microbes. Besides, NPM analyses in this study was based on NPA from hospitalized children in the RW and IC groups and FNPS from children in the CC group. There has not been any study that compared microbiome compositions in these upper airway samples, so it is unknown if such might introduce bias in the bacterial community. A previous local study revealed that these two sampling methods had comparable power to detect respiratory viruses by molecular assays (Chan et al., 2008). Further comparative studies are required to analyze upper airway microbiota in NPA and FNPS samples.

## Conclusion

We identified *M. catarrhalis* and *D. pigrum* to be the predominant species in nasopharynx of pre-school children. Hospitalized children with RW and positive for S-API had lower nasopharyngeal biodiversity than CC, while Proteobacteria was the only representative taxa in RW. On the other hand, *D. pigrum* was overrepresented in CC. Adjusted for HRV status, *D. pigrum* and five MetaCyc pathways were discriminative across groups. These microbial biomarkers provide insights into RW pathogenesis in pre-school children.

## Data Availability Statement

The datasets presented in this study can be found in online repositories. The names of the repository/repositories and accession number(s) can be found in the article/Supplementary Material.

## Ethics Statement

The studies involving human participants were reviewed and approved by the Joint Chinese University of Hong Kong – New Territories East Cluster Clinical Research Ethics Committee. Written informed consent to participate in this study was provided by the participants’ legal guardian/next of kin.

## Author Contributions

TL obtained research funding, designed this study, collected clinical data, and drafted this manuscript. YS collected clinical data, performed laboratory experiments, analyzed laboratory and WGS data, and drafted this manuscript. JH and JK conducted bioinformatics analysis, interpreted WGS data, and contributed to the manuscript. ST supervised bioinformatic data analysis and results interpretation. HW and MW contributed as statistical consultation. MT contributed to laboratory experiments. KT and RC conducted experiments for HRV detection. AL and GW analyzed clinical data. All authors reviewed and approved the manuscript.

## Conflict of Interest

The authors declare that the research was conducted in the absence of any commercial or financial relationships that could be construed as a potential conflict of interest.

## Publisher’s Note

All claims expressed in this article are solely those of the authors and do not necessarily represent those of their affiliated organizations, or those of the publisher, the editors and the reviewers. Any product that may be evaluated in this article, or claim that may be made by its manufacturer, is not guaranteed or endorsed by the publisher.

## Abbreviations

CC: community control
IC: inpatient control
RW: recurrent wheeze
HRV: human rhinovirus
CCC: community control negative for HRV
CCV: community control with HRV
ICC: inpatient control negative for HRV
ICV: inpatient control with HRV
RWV: recurrent wheezing with HRV
FNPS: flocked nasopharyngeal swab
NPA: nasopharyngeal aspirate
NPM: nasopharyngeal microbiome
SDI: Shannon diversity index
URTI: upper respiratory tract infection
WGS: whole genome shotgun.
