# Supplementary material for: Whole-Genome Shotgun Sequencing for Nasopharyngeal Microbiome in Pre-school Children With Recurrent Wheezing
Source: Front Microbiol. 2022 Feb 16;12:792556. doi: 10.3389/fmicb.2021.792556 (PMC8889122; doi:10.3389/fmicb.2021.792556)

**ONLINE SUPPLEMENT**

**PATIENTS AND METHODS**

**High-throughput sequencing and sequence data pre-processing**

WGS sequencing was performed on Illumina HiSeq X Ten platform (Illumina, San Diego, CA) to generate raw output of 93.51 giga nucleotides of paired-end reads (2 x 150 bp). The mean (s.d.) metagenomes averaged 15,392,722 (5,915,150) reads and 414,540 (582,519) per sample before and after prior to quality filtering respectively. For quality control, raw reads were pre-processed using KneadData version 0.7.2^S1^, which utilized Trimmomatic version 0.36^S2^ trimming and Bowtie2 version 2.3.4.3 decontamination algorithms^S3^ to remove low quality read bases and human DNA sequences, respectively. Specifically, Trimmomatic was first run with parameters of 4-base wide sliding window and an average Phred score threshold of 20. Trimmed non-human reads shorter than 50 nt were discarded, and quality-filtered reads were further aligned against human reference genome GRCh37/hg19 to filter for host sequence decontamination.

**Taxonomic and functional analysis**

The resulting non-human reads were taxonomically profiled using MetaPhlAn2^S4^ version 2.7.7, a reference-based taxonomic classifier, to determine the microbial composition in the dataset. This computational tool maps non-human reads to a database of ~1 million unique clade-specific maker genes and estimates the relative abundances of each microbe present in samples to species-level resolution. Those clade-specific markers, encompassing ~13,500 bacterial and archaeal, ~3,500 viral and ~110 eukaryotic reference genomes, are preselected from coding sequences that allows unequivocal identification of specific microbial clades at species or higher level.

Functional pathways were profiled using HUMAnN2^S5^ version 0.11.2 with defaulting parameters. HUMAnN2 exploits a ‘three-tier search’ strategy to determine the function content of a metagenome at species level. Firstly, MetaPhlAn2 taxonomic profile were used to construct sample-specific pangenomes of all species present in a sample. Next, HUMAnN2 implements a nucleotide alignment of all sample reads onto the sample’s pangenome database using bowtie2^S2^, which provides a quick explanation of most of the reads. Finally, the unmapped reads were subjected to comprehensive translated search against UniRef90^S6^ protein database using DIAMOND^S7^. Hits were counted and normalized for each gene family, and the latter was then combined into pathways based on both the HUMAnN2 default MetaCyc^S8^.

To visualize the results, heatmaps of relative abundances for bacteria and viruses and boxplots of Shannon index were generated using R version 3.5.0 packages ComplexHeatmap (clustering rows and columns based on Bray-Curtis dissimilarity) and ggplots2v, respectively.

**Statistical analysis**

ANOVA test and χ^2^ test were used to compare continuous and categorical variables respectively, and Wilcoxon rank-sum test was used to analyse differences between two groups and Kruskal-Wallis test for those among three or more groups. Shannon index was used to compare microbial richness and evenness (i.e. alpha diversity) between sample groups. Regarding taxonomic and functional profiles, Statistical Analysis Metagenomic Profiles (STAMP)^S9^ software version 2.0 was used to compare between-group differences based on ANOVA with Games-Howell post-hoc test. Storey false discovery rate (FDR) was used to correct for multiple testing. STAMP also outputs visualized graphs for statistical results with effect size for each feature that indicate their assessed biological importance. Linear discriminant analysis effect size (LEfSe) algorithm^S10^ was applied to identify discriminating features that are not only statistically but also biologically significant (i.e. biomarker discovery). The cut-off criteria were set as default unless noted otherwise. Non-parametric Kruskal-Wallis rank sum test was used to analyse differences between classes (i.e. three clinical features RW, CC and IC), and pairwise Wilcoxon test was used to test differences between subclasses (i.e. HRV infection status) of different classes. Logarithmic LDA score >2 was set as the threshold to estimate effect size, and one-against-all strategy was selected for multi-class analysis.

Considering the complexity of metagenomic profiles, it is important to reproduce the results before we can draw any conclusion^S11,S12^. Hence, analysis of composition of microbiomes (ANCOM)^S13^, a test with sensitivity on complexed microbiome profile while controlling for FDR, was further performed to verify the reliability of findings from LEfSe analysis. ANCOM tests the null hypothesis that the taxon has the same mean abundance on average at the ecosystem level. It generates test statistics based on compositional log-ratios and is capable of adjusting for covariates under a linear model framework^S14^. Considering the impact of HRV on microbial components^S15,S16^, HRV infection status was also included as a confounder in our data analysis. This study considered only those microbes and pathways displaying consistent significance from all three analyses as well as surviving the adjustment for HRV status as being significant. *P* < .05 was set as the significance threshold.

**SUPPLEMENTARY REFERENCES**

S1. The Huttenhower Lab. KneadData. Available online: http://huttenhower.sph.harvard.edu/kneaddata [accessed on 18 Dec 2018].

S2. Bolger AM, Lohse M, Usadel B. Trimmomatic: a flexible trimmer for Illumina sequence data. *Bioinformatics*. 2014;30(15):2114-2120.

S3. Langmead B, Salzberg SL. Fast gapped-read alignment with Bowtie 2. *Nat Methods*. 2012;9(4):357.

S4. Truong DT, Franzosa EA, Tickle TL, et al. MetaPhlAn2 for enhanced metagenomic taxonomic profiling. *Nat Methods*. 2015;12(10):902-903.

S5. Franzosa EA, McIver LJ, Rahnavard G, et al. Species-level functional profiling of metagenomes and metatranscriptomes. *Nat Methods*. 2018;15(11):962-968.

S6. Suzek BE, Huang H, McGarvey P, Mazumder R, Wu CH. UniRef: comprehensive and non-redundant UniProt reference clusters. *Bioinformatics*. 2007;23(10):1282-1288.

S7. Buchfink B, Xie C, Huson DH. Fast and sensitive protein alignment using DIAMOND. *Nat Methods*. 2015;12(1):59-60.

S8. Caspi R, Billington R, Keseler IM, et al. The MetaCyc database of metabolic pathways and enzymes - a 2019 update. *Nucleic Acid Res*. 2020;48(D1):D445-D453.

S9. Parks DH, Tyson GW, Hugenholtz P, Beiko RG. STAMP: statistical analysis of taxonomic and functional profiles. *Bioinformatics*. 2014;30(21):3123-3124.

S10. Segata N, Izard J, Waldron L, et al. Metagenomic biomarker discovery and explanation. *Genome Biol*. 2011;12(6):R60.

S11. Dhariwal A, Chong J, Habib S, et al. MicrobiomeAnalyst: a web-based tool for comprehensive statistical, visual and meta-analysis of microbiome data. *Nucleic Acid Res*. 2017;45(W1):W180-188.

S12. Koohi-Moghadam M, Borad MJ, Tran NL, et al. MetaMarker: a pipeline for de novo discovery of novel metagenomic biomarkers. *Bioinformatics*. 2019;35(19):3812-3814.

S13. Mandal S, Van Treuren W, White RA, et al. Analysis of composition of microbiomes: a novel method for studying microbial composition. *Microbial Ecol Health Dis*. 2015;26:27663.

S14. Weiss S, Xu ZZ, Peddada S, et al. Normalization and microbial differential abundance strategies depend upon data characteristics. *Microbiome*. 2017;5(1):27.

S15. Molyneaux PL, Mallia P, Cox MJ, et al. Outgrowth of the bacterial airway microbiome after rhinovirus exacerbation of chronic obstructive pulmonary disease. *Am J Respir Crit Care Med*. 2013;188(10):1224-1231.

S16. Korten I, Mika M, Klenja S, et al. Interactions of respiratory viruses and the nasal microbiota during the first year of life in healthy infants. *mSphere*. 2016;1(6).pii:e00312-16.

**TABLE S1** Clinical characteristics of study participants

|  | RW (n=16) | IC (n=18) | | CC (n=36) | | P-value |
| --- | --- | --- | --- | --- | --- | --- |
| Respiratory symptoms | Wheezing | URTI | | None | | - |
| History of asthma | Allergic asthma | Never | | Never | | - |
| Hospitalization status | Inpatient | Inpatient | | Not hospitalized | | - |
| HRV status | HRV+ | HRV+ | HRV- | HRV+ | HRV- | 3.1e-6 |
| Number of samples | 16 | 6 | 12 | 8 | 28 | - |
| Mean (SD) age, year | 3.6 (1.0) | 3.5 (1.0) | | 4.9 (0.6) | | 3.6e-8 |
| Male (%) | 11 (68.8) | 11 (61.1) | | 18 (50.0) | | 0.225 |

Kruskal-Wallis test or Chi-Square test was performed as appropriate.

Abbreviations: RW, recurrent wheeze; CC, community control; HRV, human rhinovirus; IC, inpatient control; URTI, upper respiratory tract infection; +, positive; -, negative.

**TABLE S2** Top 11 most abundant species and their 90^th^ percentile RA across all samples

| Species | Genus | Phylum | RA (%) |
| --- | --- | --- | --- |
| *Moraxella catarrhalis* | *Moraxella* | *Proteobacteria* | 75.69 |
| *Dolosigranulum pigrum* | *Dolosigranulum* | *Firmicutes* | 73.86 |
| *Porcine type C oncovirus* | *Gammaretrovirus* | N/A* | 12.29 |
| *Streptococcus mitis oralis pneumoniae* | *Streptococcus* | *Firmicutes* | 10.73 |
| *Haemophilus influenzae* | *Haemophilus* | *Proteobacteria* | 9.66 |
| *Corynebacterium pseudodiphtheriticum* | *Corynebacterium* | *Actinobacteria* | 9.62 |
| *Escherichia unclassified* | *Escherichia* | *Proteobacteria* | 8.01 |
| *Bifidobacterium breve* | *Bifidobacterium* | *Actinobacteria* | 7.22 |
| *Akkermansia muciniphila* | *Akkermansia* | *Verrucomicrobia* | 7.19 |
| *Bifidobacterium longum* | *Bifidobacterium* | *Actinobacteria* | 2.41 |
| *Escherichia coli* | *Escherichia* | *Proteobacteria* | 1.13 |

Abbreviation: RA, relative abundance.

* N/A: phylum of *Gammaretrovirus* remains incertae sedis.

**TABLE S3** Summary of 33 species (21 bacteria, 11 viruses, and one eukaryote) with different relative abundances among RW, IC, and CC groups.

*This Table can be found in the corresponding Excel file*.

**TABLE S4** Summary of top 20 most abundant pathways as ranked by 90^th^ percentile of relative abundance across all samples.

*This Table can be found in the corresponding Excel file*.

**TABLE S5** STAMP analyses for the functional potential of NPM that identified 32 pathways to be significantly different between groups. Twenty-two of them were responsible for biosynthesis of essential life components for bacteria including nucleoside and nucleotide, amino acid, fatty acid and peptidoglycan. Seven of these pathways fell into the “generation of precursor metabolite and energy” category. Finally, three pathways belonged to “Degeneration/utilization/assimilation” category.

*This Table can be found in the corresponding Excel file*.

**TABLE S6** Nine MetaCyc pathways that were predicted to involve *Dolosigranulum pigrum*

| MetaCyc ID | Name of pathway | Abundance by *D. pigrum* (mean ± SD) |
| --- | --- | --- |
| COA-PWY-1 | Coenzyme A biosynthesis II (mammalian) | 0.00184 ± 0.00566 |
| NONOXIPENT-PWY | Pentose phosphate pathway (non-oxidative branch) | 0.00183 ± 0.00378 |
| PEPTIDOGLYCANSYN-PWY | Peptidoglycan biosynthesis I (meso-diaminopimelate containing) | 0.00142 ± 0.00364 |
| PWY-6151 | S-adenosyl-L-methionine cycle I | 0.00402 ± 0.00815 |
| PWY-6386 | UDP-N-acetylmuramoyl-pentapeptide biosynthesis II (lysine-containing) | 0.00169 ± 0.00428 |
| PWY-6387 | UDP-N-acetylmuramoyl-pentapeptide biosynthesis I (meso-diaminopimelate containing) | 0.00148 ± 0.00366 |
| PWY-7219 | Adenosine ribonucleotides de novo biosynthesis | 0.00700 ± 0.01473 |
| PWY-7221 | Guanosine ribonucleotides de novo biosynthesis | 0.00199 ± 0.00399 |
| PWY0-1296 | Purine ribonucleoside degradation | 0.00340 ± 0.00990 |

**FIGURE S1** Alpha and beta diversity of NPM in 5 subgroups stratified by subjects’ HRV status. (A) Shannon diversity index differs across subgroups. (B) Principal coordinate analysis (PCoA) plot based on Bray-Curtis dissimilarity. Points refer to samples that were colour coded by each subgroup. PERMAOVA test were performed and overall P-value was shown. CCC, community control negative for HRV; CCV, community control with HRV; ICC, inpatient control negative for HRV; ICV, inpatient control with HRV; RWV, recurrent wheezing with HRV.

**(A)**


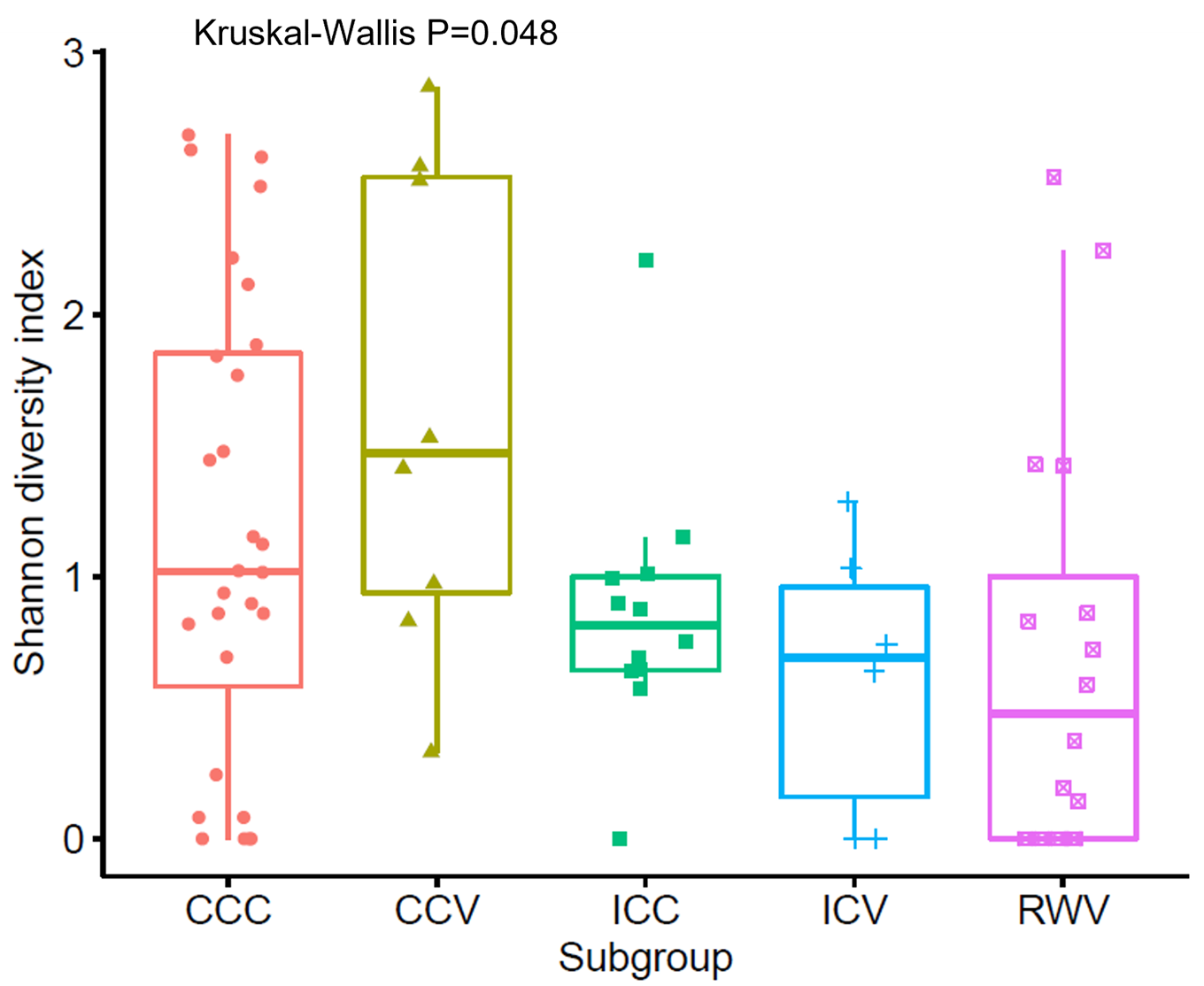


**(B)**


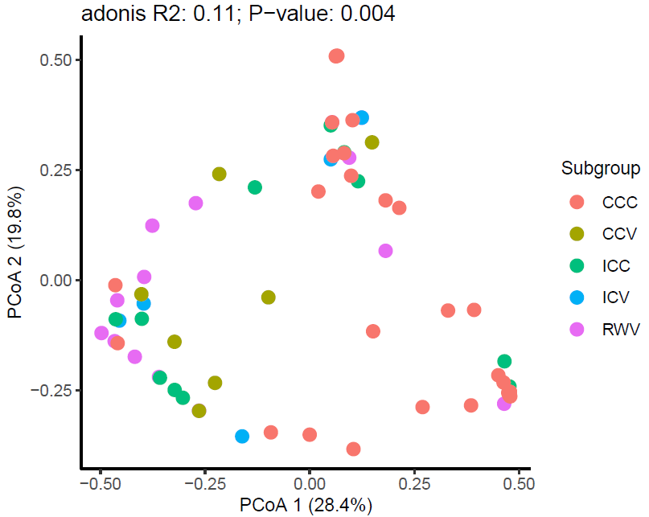


**FIGURE S2** Heatmap of relative abundances of all 41 species in individual samples ordered by bacterial, viral, and eukaryotic kingdoms from the top to the bottom. All samples were hierarchically clustered based on Bray-Curtis dissimilarity of species abundances. Samples were colour coded according to groups and HRV status as indicated. CC, community control; IC, inpatient control; RW, recurrent wheeze.


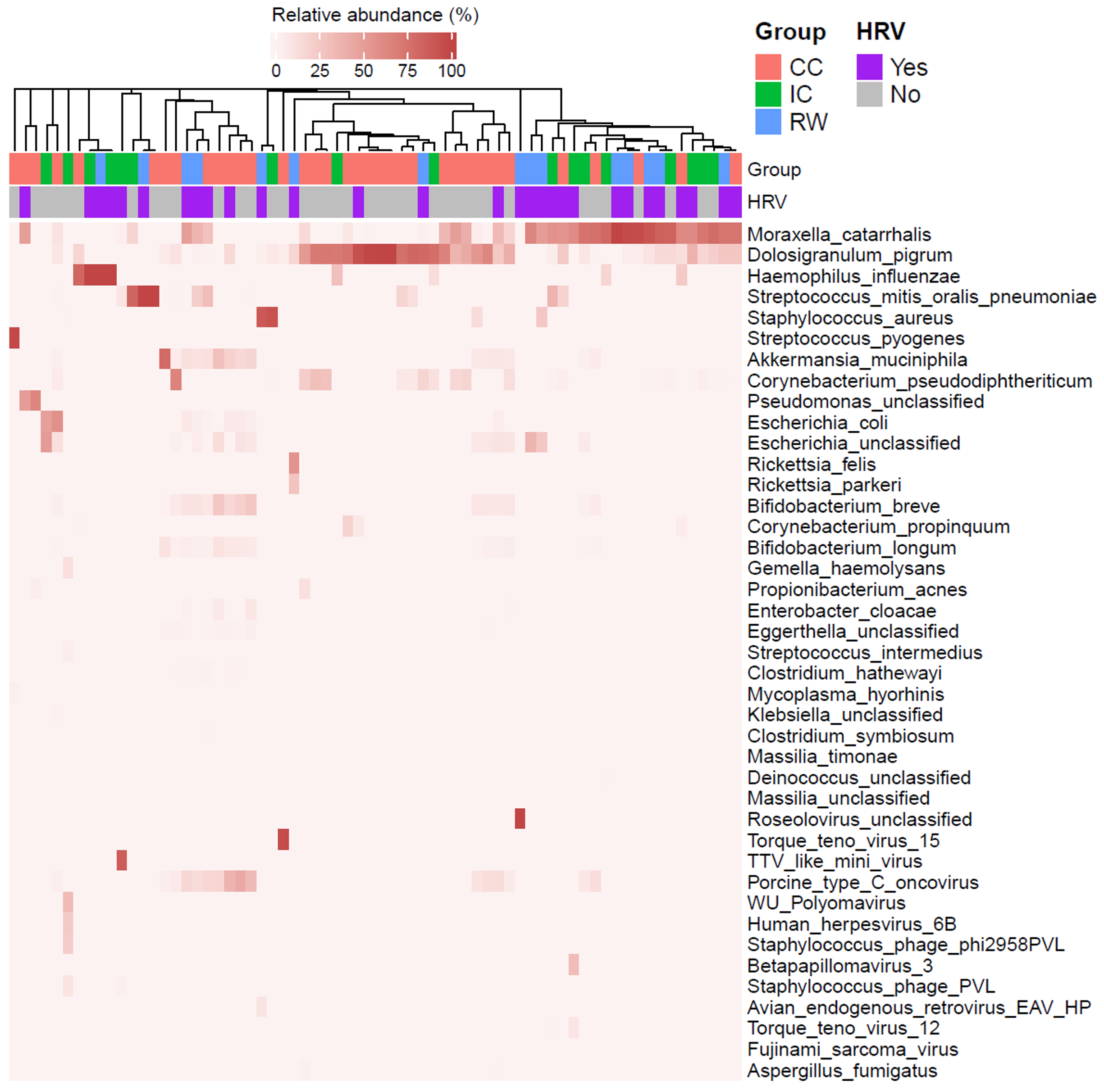


**FIGURE S3** Relative abundance of *porcine type C oncovirus* in five subgroups


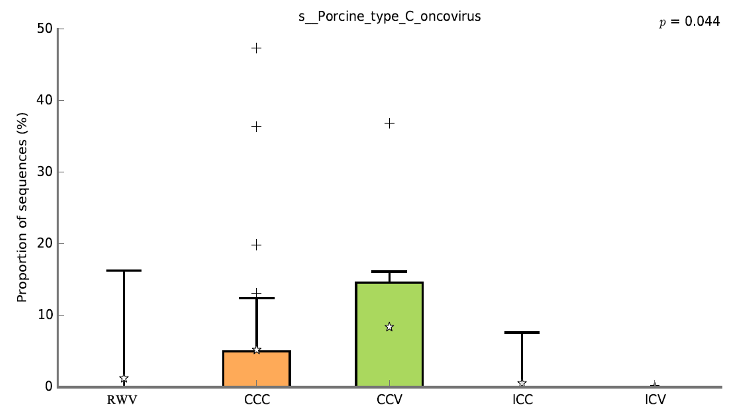


Abbreviations: RWV, recurrent wheezing with HRV; ICV, inpatient control with HRV; ICC, inpatient control without HRV; CCV, community control with HRV; CCC, community control without HRV.

**FIGURE S4** Histogram showing all taxa that were significantly more abundant in CC group and survived the “biological consistency” assumption in LEfSe analysis. Prefixes refer to abbreviations of taxonomic rank for each taxon, with phylum (p_), class (c_), family (f_), genus (g_), species (s_) and strain (t_).


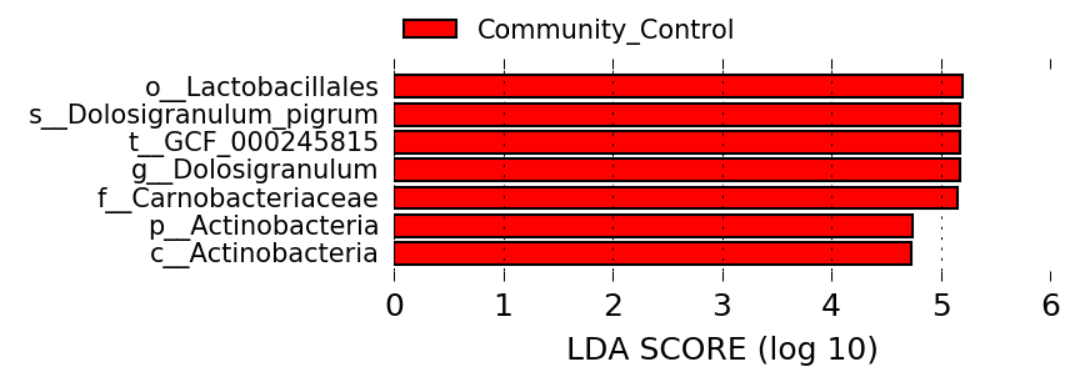


**FIGURE S5** Discriminative taxa in NP microbiome identified by LEfSe analyses among RW patients and controls. (A) Histogram of discriminative microbes between RW (red) and CC (green). Taxa shown in the histogram were statistically significant (*P* < .05). Prefixes refer to abbreviations of taxonomic rank for each taxon, with phylum (p_), class (c_), family (f_), genus (g_), species (s_) and strain (t_). (B) Taxonomic cladogram of the taxa mentioned in Fig. 3A, with taxa enriched in RW in red and CC in green. The same coding scheme for taxonomic rank as Figure 3A was used. Yellow spots indicate taxa with no significantly discriminative features. Size of the node is proportional to log of relative abundance for the corresponding taxon. (C) Histogram showing the relative abundance of *D. pigrum* in RW, IC and CC groups. Red bars indicate samples positive for HRV while green bars represent those negative for HRV. Solid and dashed lines indicate mean and median values respectively. Cases is RW; Community is CC; URI is IC.

**(A)**


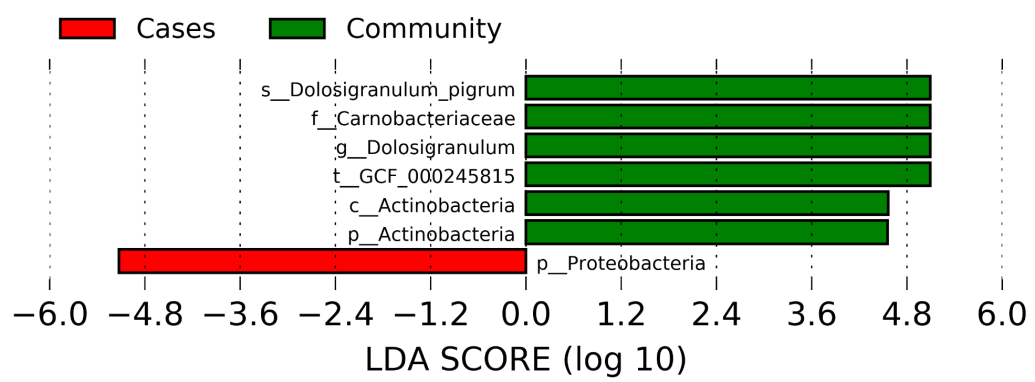


**(B)**


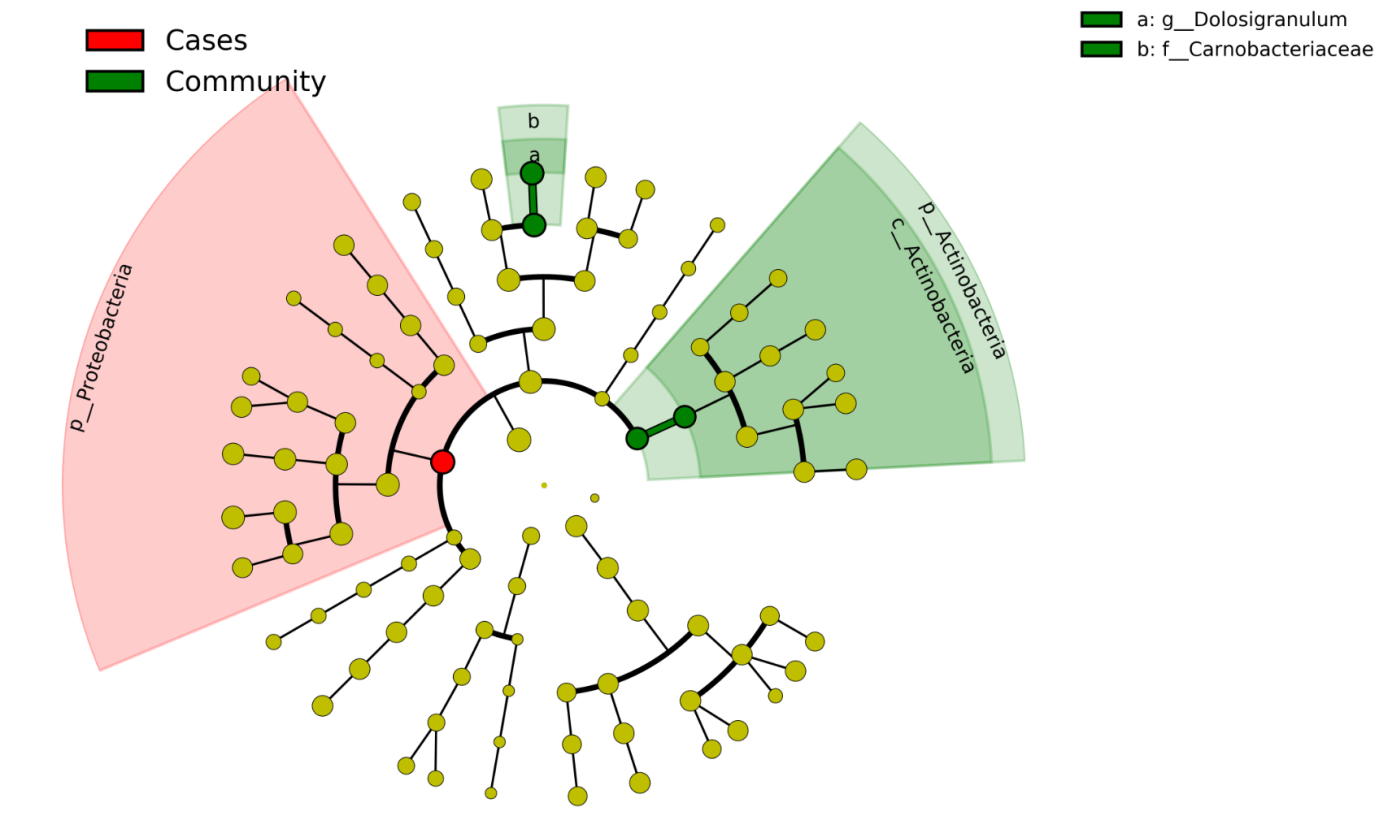


**(C)**


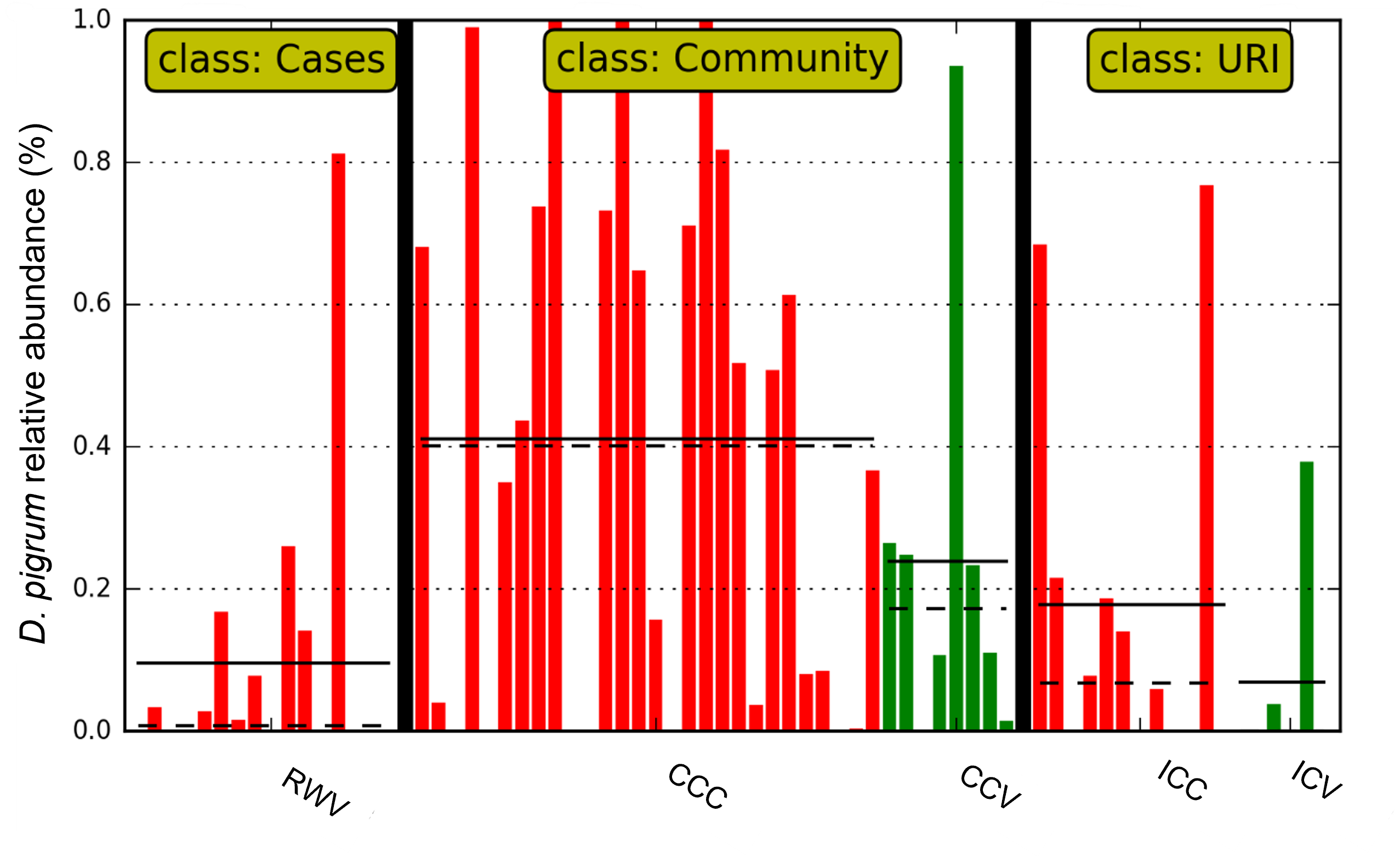


**FIGURE S6** Boxplots showing the relative abundances of significantly differentially abundant taxa identified by ANCOM after controlling for age and HRV status as covariate. (A) Left panel, *D. pigrum* species had higher relative abundance in CC. (B) Middle panel, the relative abundance of *H. influenzae* species was no longer significantly differentially abundant across groups when controlling for HRV status. (C) Right panel, Proteobacteria was more abundant in RW than controls regardless of HRV status.


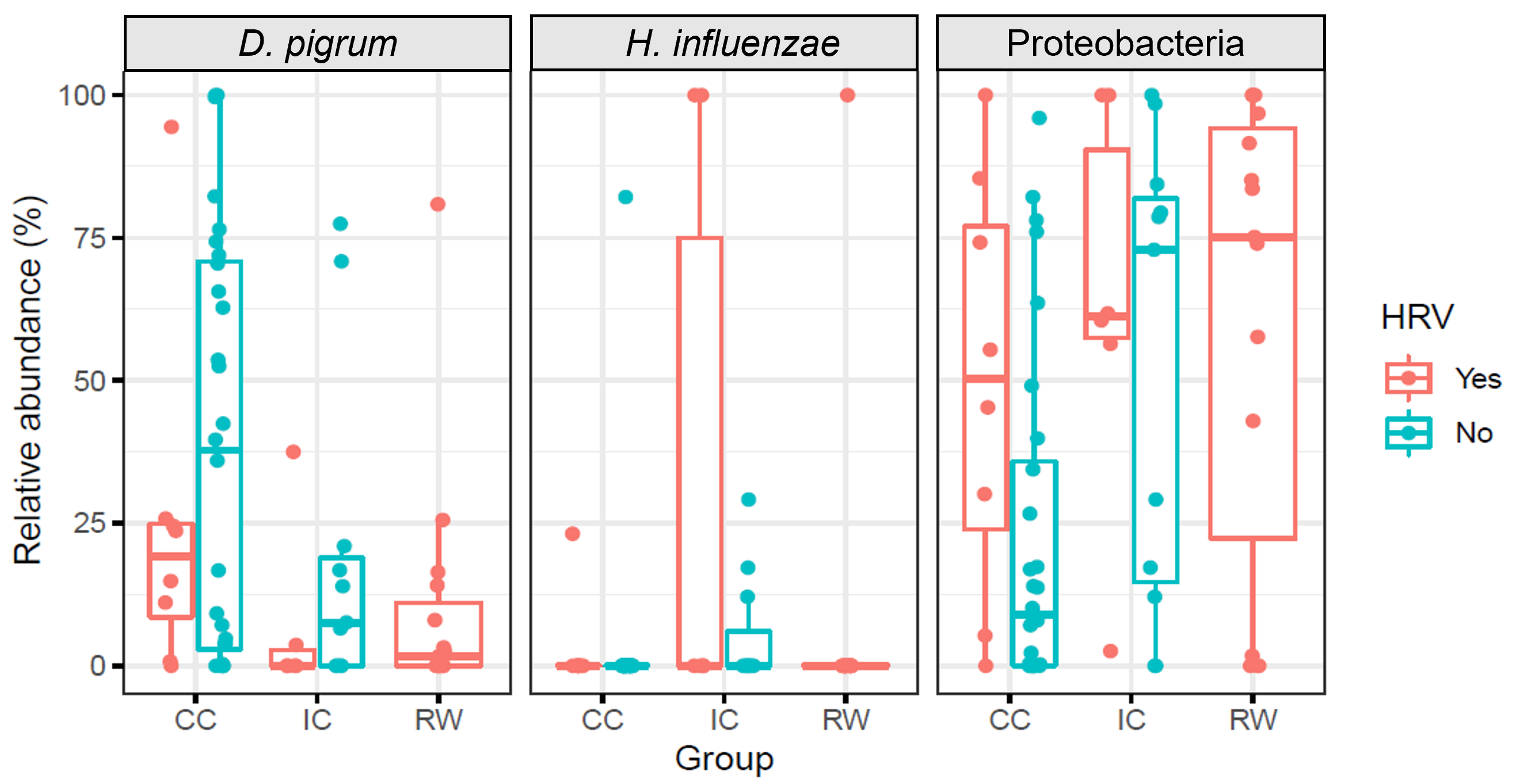


**FIGURE S7** Contributional diversity of discriminative pathways by LEfSe analysis. Stacked barplots showed the contributional diversity and relative abundance of pathways across samples for (A) adenosine nucleotides de novo biosynthesis I; (B) adenosine nucleotides de novo biosynthesis II; (C) guanosine nucleotides de novo biosynthesis II; and (D) adenosine ribonucleotides de novo biosynthesis. Only samples with non-zero relative abundances were shown. CCC, community control negative for HRV; CCV, community control with HRV; ICC, inpatient control negative for HRV; ICV, inpatient control with HRV; RWV, recurrent wheezing with HRV.

**(A)**


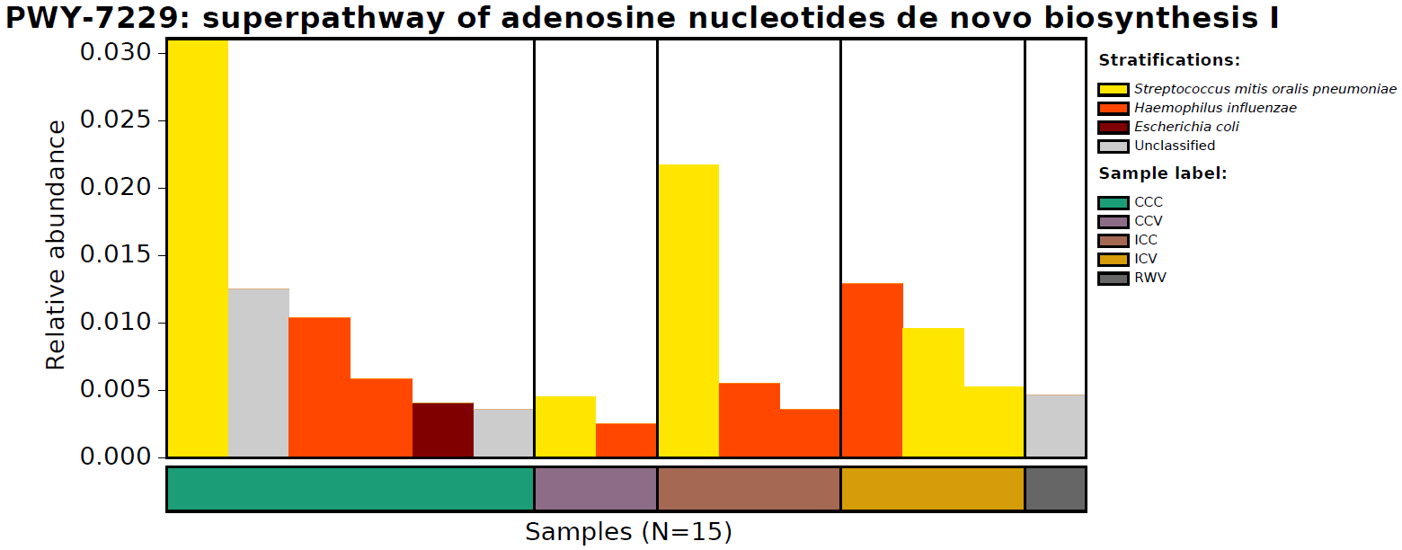


**(B)**


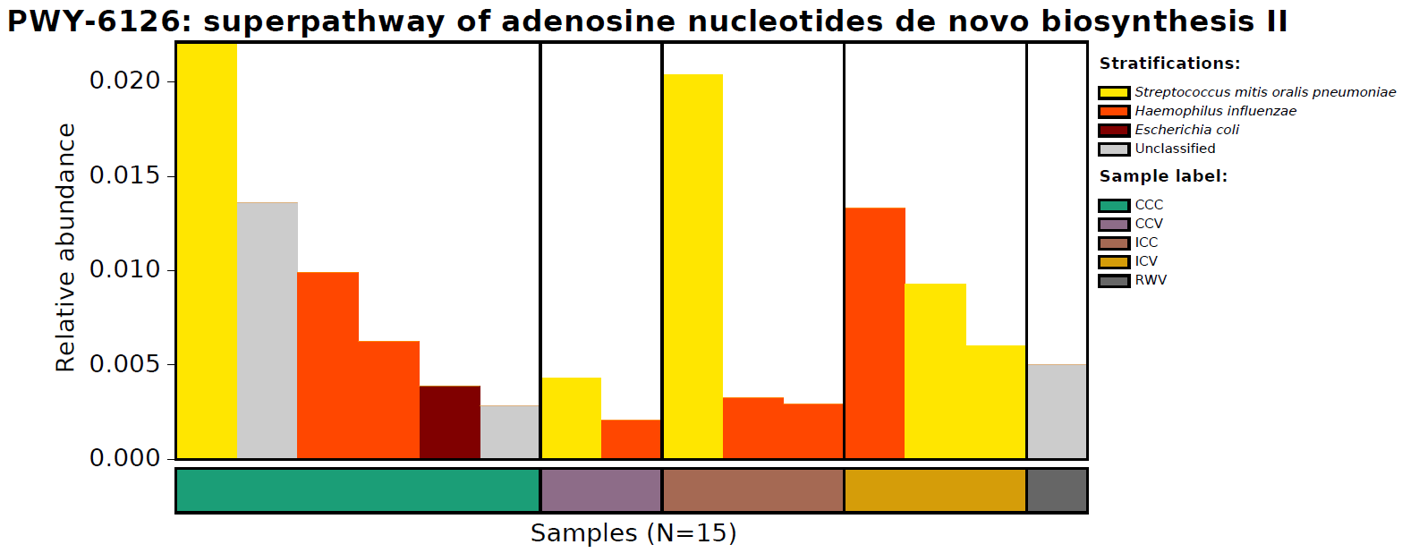


**(C)**


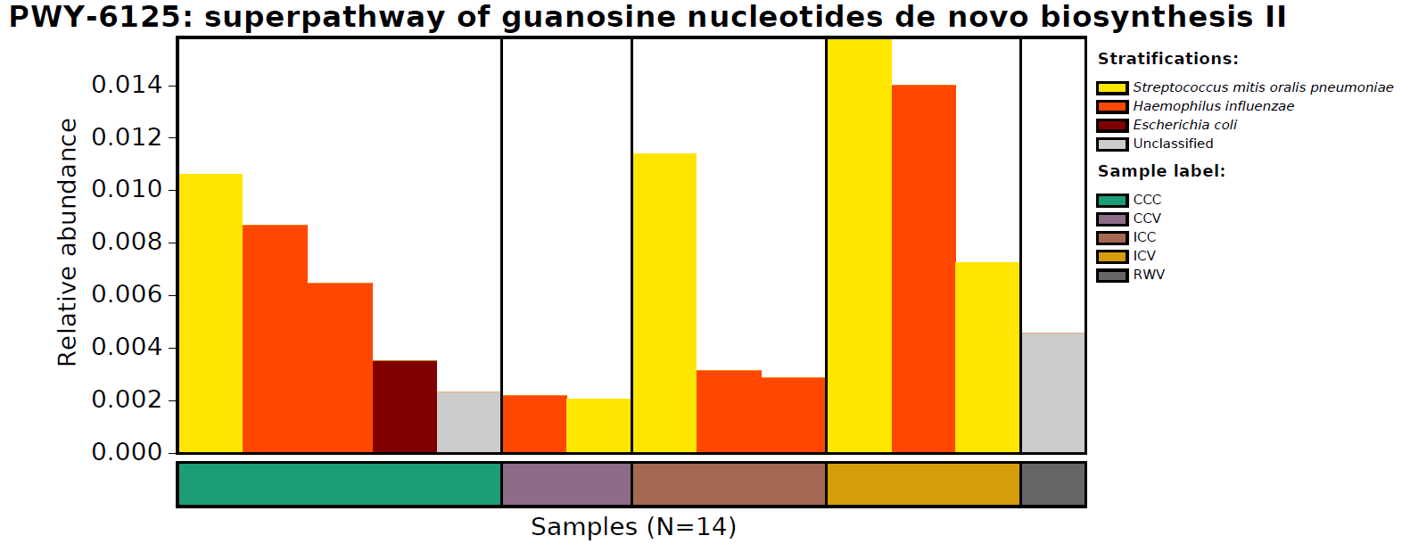


**(D)**


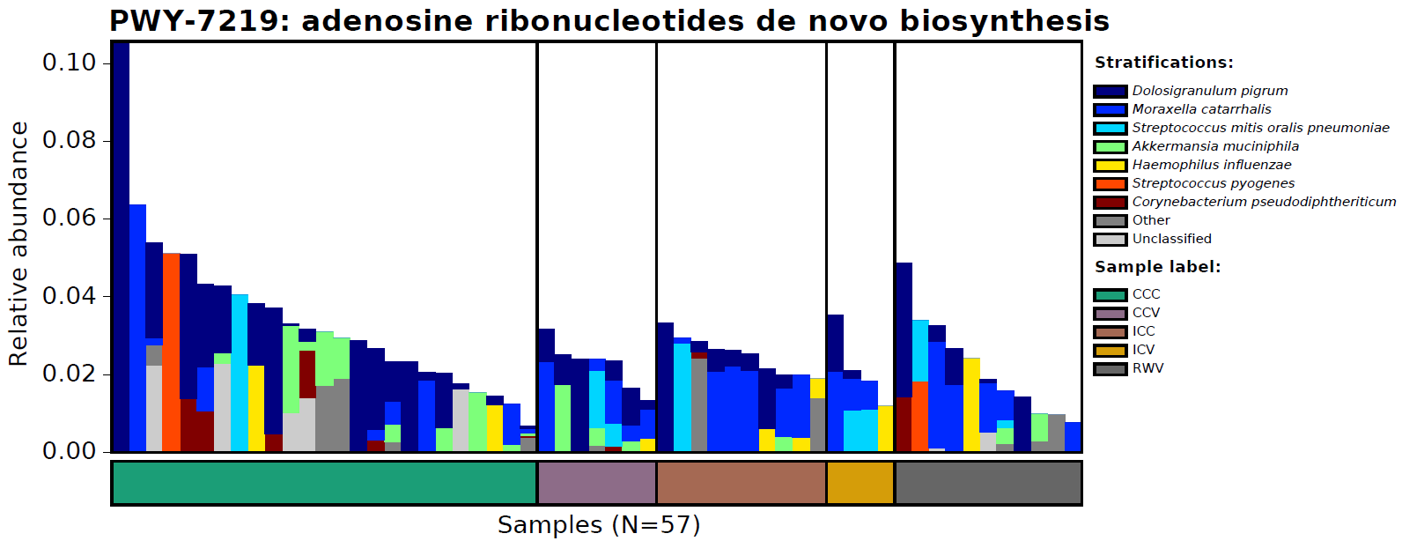


**FIGURE S8** Contributional diversity of the top pathways (A) S-adenosyl-L-methionine cycle I and (B) purine ribonucleosides degradation that were assumed to involve *D. pigrum*. Only samples with non-zero relative abundances were shown. CCC, community control negative for HRV; CCV, community control with HRV; ICC, inpatient control negative for HRV; ICV, inpatient control with HRV; RWV, recurrent wheezing with HRV.

**(A)**


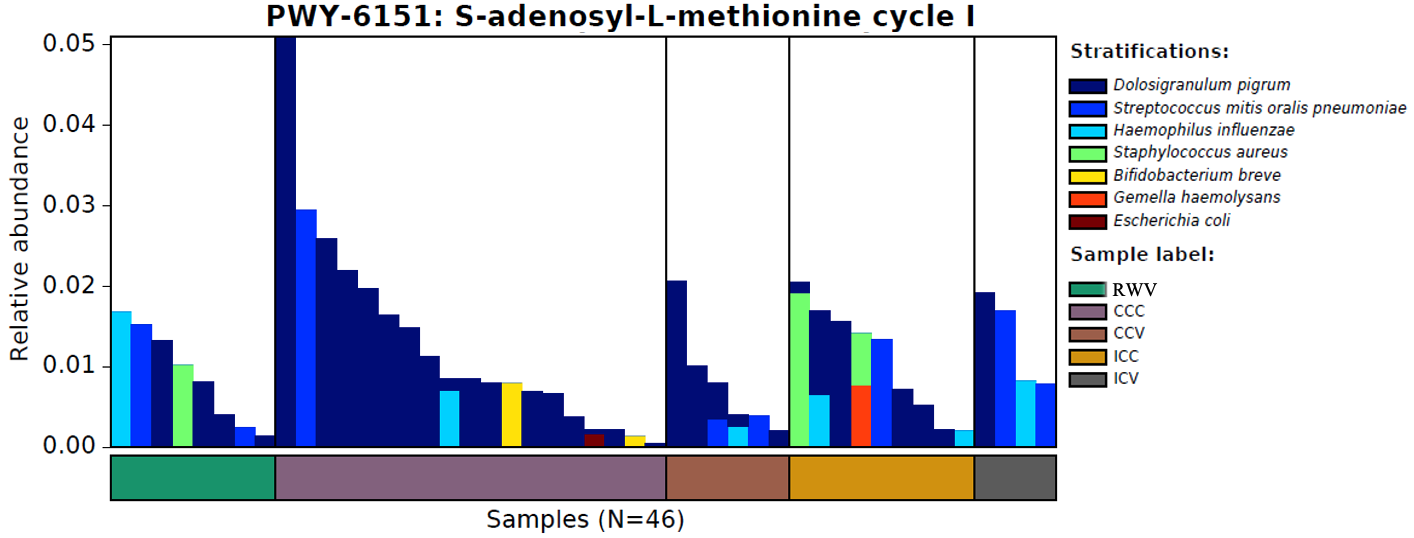


**(B)**


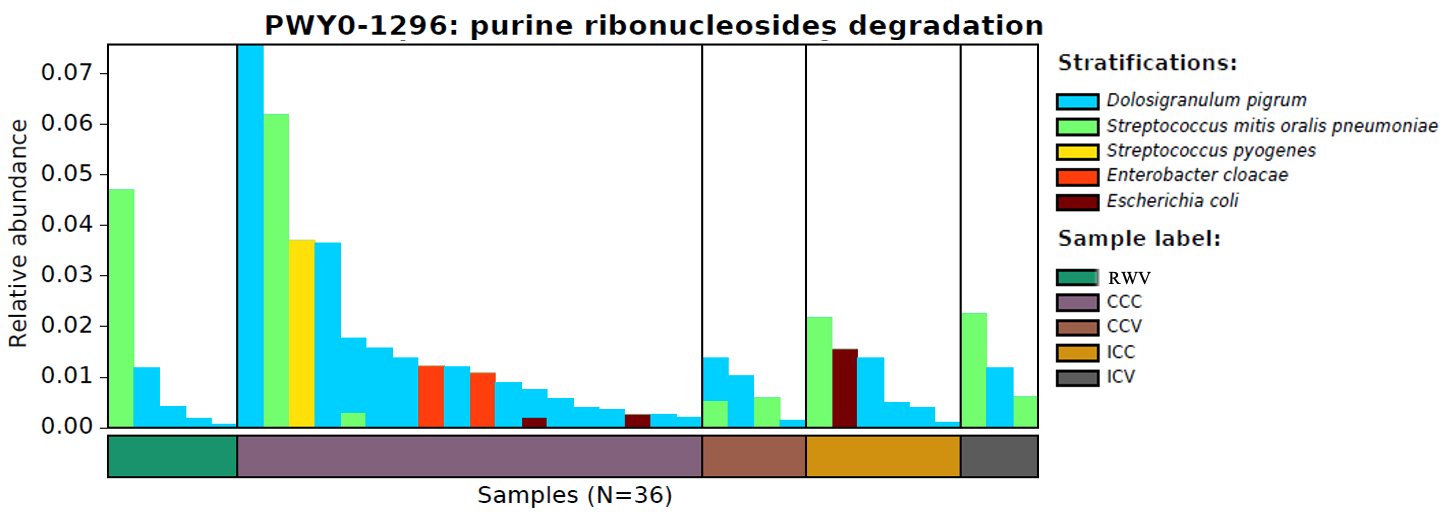

Supplement: Supplementary file 1 [file Data_Sheet_1.docx]
